# 

**Published:** 1874-12

**Authors:** 


*Vol. XXVIII.* **DECEMBER, 1874.** *No. 12.*

for J. S. Billings

### THE DENTAL REGISTER,

A Monthly Journal Devoted to the Interests of the Profession.

EDITED BY J. TAFT, D. D. S.

AND

PUBLISHED BY SPENCER, CROCKER & CO.,
OHIO DENTAL DEPOT.
168 Race Street, Cincinnati, Ohio.

TERMS: $2.50 Per Annum, in Advance; Single Copies 25c.

J. P. GEPPERT, PRINTER

